# Band 5 Nurses' Leadership Development as a Current Care Priority in England: A Qualitative Study of Perceptions, Barriers, and Ways Forward

**DOI:** 10.1155/2023/3145908

**Published:** 2023-07-21

**Authors:** Tony Conner, Mark Parkinson, Juliana Thompson

**Affiliations:** Northumbria University, Newcastle-upon-Tyne, UK

## Abstract

**Aims:**

To explore band 5 staff nurses' perceptions of leadership and leadership development and derive insights and recommendations to inform future practice.

**Background:**

Band 5 staff nurses are increasingly expected to develop leadership skills but are not always well supported in this.

**Method:**

A qualitative methodology situated within a constructivist paradigm explored the shared meanings and understandings of band 5 nurses' leadership development within the context of current organisation, policy, and culture.

**Results:**

Three principal themes representing band 5 nurses' perceptions of leadership were identified: defining leadership, opportunities to lead, promoting leadership development.

**Conclusion:**

Band 5 nurses' leadership development is highly variable in frequency and quality. Key barriers and facilitators to development are discussed, including the wide provision of formally validated and bespoke leadership programmes that combine practice-based, informal training. *Implications for Practice*. Nurse leadership development at all levels remains integral to high-quality and safe health care. High rates of senior staff attrition and recent guidelines in England reinforce the need for band 5 nurse leadership. The multiple challenges impeding this are discussed, alongside ways of overcoming them.

## 1. Background

In England, there is at present a relatively heavy emphasis on nurses' development of leadership skills [1] to ensure the care nurses provide and delegate is person-centred and of a consistently high standard [1], and to act as a role model for others [1]. The NHS Healthcare Leadership model [2] emphasises how all individuals in a healthcare organisation can develop their leadership skills, not only those with formal leadership roles. Current workforce policy, therefore, requires all nurses to develop leadership skills, irrespective of band or grade. A strong relationship has been found to be linked between nurses' leadership skills and patient outcomes [3], including that good nursing leadership supports the provision of high-quality care [1, 2, 4, 5], improvements in quality of care provided [6], patient safety [7, 8], patient-centred care [9], patient satisfaction [10], staff recruitment, retention and job satisfaction [11, 12], effective change management [13], costs reduction [14, 15], and financial performance [16].

Even newly qualified nurses are expected to lead on a day-to-day basis; however, they often lack the confidence to lead [17]. Nurses who are qualified for university degree level enter the NHS at band 5 and are expected to provide excellent professional, skilled, and effective person-centred, evidence-based nursing care [18]. At the current pay scale, band 5 nurses earn £27,055 to £32,934 [19]. As they gain further knowledge, skills, and experience, they may progress to higher bands within the nursing banding system [20].

Arguably, the current situation in England in which newly qualified nurses often do not receive the training they require to develop their leadership skills early in their careers, risks leaving a crucial skills gap that may extend into the longer term, stymying important leadership development and losing a valuable opportunity to enable nurses to further improve the quality of their patient care [17]. Conversely, there are multiple benefits associated with proactively identifying and developing internal candidates, yet fewer than 7% of healthcare organisations have implemented formal leadership succession planning programmes [14].

A strong argument exists therefore for prioritising nurse leadership training, with potential benefits for individuals and organisations, with “the need for leadership at all levels of nursing… overwhelmingly evident” [21]. Despite this, clinical leadership training has been predominantly focused on managers and management-focused training [22], a situation which has historically excluded band 5 nurses from leadership training [23] with a negative impact on workforce effectiveness, efficiency, and quality of care delivery [24]. Transitioning from band 5 to band 6 arguably demands high levels of leadership skills, and band 5 nurses may not possess which has led to calls for immediate action to prepare a new generation of nursing leaders [25]. Such calls take on greater urgency given high levels of attrition among experienced nurses in England, who are either leaving the profession or widely anticipated to through a combination of age (1 : 5 nurses in England are 56 years of age or over) and escalating work pressures [26].

Moreover, care service and quality demonstrate improvements where health professionals *at all levels* have access to appropriate leadership education [22]. Current leadership programmes tend to prepare nurses for leadership roles only *after* promotion [27], with scant opportunity to develop skills in advance. This may hamper leadership development at band 5 and impede the transition to higher levels within the service.

## 2. Leadership in Nursing

Despite current requirements for all nurses to develop leadership skills [1], there remains a lack of consensus regarding how “leadership” should be defined in nursing [28]. The essence of leadership includes nurses' ability to influence and direct patient care by demonstrating exemplary care [29]. However, leading care is not directly synonymous with leading other staff [30] although there may be much overlap between the two.

Additionally, there is a lack of consistency concerning the styles of leadership employed by senior staff that can be a pivotal role in inspiring band 5 nurses' own leadership development. Winston and Patterson [31] identified in excess of 90 dimensions of leadership which demonstrates the complexity of leadership as a concept. These can be combined and contribute to leadership styles in highly variable ways that may range along a spectrum from autocratic to transformational [32, 33].

While taxonomies detailing key attributes of leadership exist (e.g., NHS [2]), it is less clear how band 5 nurses themselves perceive leadership or opportunities to lead, how different perceptions emerge, how these may differ depending on different settings and circumstances, and which specific contexts currently contribute to leadership development or impede it. Leadership and how it is defined and practiced is likely to vary according to different care settings, bands, and grades that calls for greater specificity and a move away from using the term “leadership” too generically [34].

Although preceptorship programmes are highly valued by nurses [35], they are not a substitute for continuing leadership development [36] that is underpinned by active, structured support [37].

These issues have added gravitas given the significant attrition of nurse leaders in the UK within an ageing workforce [38] which necessitates the recruitment of younger and less experienced nurses to fill the gap. This is a situation further exacerbated by low staffing levels and high workloads during the pandemic [39] and subsequently that has led to less experienced staff having to take on significant levels of responsibility.

This study explored these issues and challenges by engaging with band 5 nurses across a diverse range of healthcare contexts, settings, and circumstances. Given the NMC's requirement for nurse leaders to manage and lead care [1] and RCN's [40] call to promote nurse leadership development to ensure high quality, safe, and compassionate health care, this represents a timely juncture to explore the issues and challenges surrounding band 5 nurses' leadership, alongside ways of overcoming them, and to use the insights gained to add to the current knowledge base.

## 3. Methods

The research team consisted of three academic staff who contributed equally to data collection and subsequent analysis. The lead researcher was an assistant professor of nursing with expertise in leadership and management. The other team members were an associate professor of nursing and a senior research assistant, both with research expertise in workforce development. The research team felt that this study's exploration of shared understandings within organisational, policy, and cultural contexts aligned well with Crotty's [41] view, “that all knowledge, and therefore all meaningful reality as such, is contingent upon human practices, being constructed in and out of an interaction between human beings and their world, and developed and transmitted within an essentially social context.” This supported the rationale for adopting a qualitative methodology situated within a constructivist paradigm.

### 3.1. Sample

To gain insights into band 5 nurses' experiences of leadership across a diverse range of contexts, the plan was to locate the study in NHS primary care, community care, secondary care, and care homes operated by private and voluntary sector providers across North East England. Participants were accessed via gatekeepers who were service managers working in these providers (matrons, GP practice managers, care home managers, and lead community nurses). Gatekeepers provided their band 5 nursing staff with study information explaining the purpose/aims of the study and what participation involved. Staff were given time to read information sheets which emphasised participation was voluntary and that deciding not to participate would not affect staff's employment in any way. Information sheets invited nurses to contact the researchers with any questions before agreeing to participate. Participants provided written consent.

In total, 18 band 5 nurses responded to the invitation to participate. All were accepted as participants in the study, and all consented to participate. The response rate was lower than predicted due to the outbreak of COVID-19. This impacted the availability of staff to participate, necessitating some scaling back of recruitment, particularly from primary care and voluntary sectors. Despite this, the remainder of the study protocol remained intact and recruitment possible from secondary care (*n* = 14), care homes (*n* = 3), and community nursing services (*n* = 1), reflecting a diverse range of nursing roles, NHS trusts, and care-provider operators (Table 1). As well as representing heterogeneity in terms of being drawn from different clinical settings, the sample was varied regarding age groups (20 to 69 years of age), sex, nursing experience from <1 year to >30 years, and country of birth.

### 3.2. Data Collection

Data were collected via individual semistructured interviews. All members of the research team conducted data collection. The first three interviews were face-to-face. Subsequent interviews were conducted online or by telephone due to COVID-19. Interview questions were informed by the findings of the literature review and explored participants' own experiences of leadership development and practice, and reasons underpinning them. Average length of interviews was 46 minutes, and none exceeded one hour. This afforded time to record participants' in-depth accounts of their experiences.

### 3.3. Data Analysis

Interviews were audio-recorded and transcribed verbatim. Thematic analysis (TA) was used to analyse the data and selected for its proficiency in organising, analysing, and reporting patterns (themes) within data in rich detail [42]. An inductive analytical approach was adopted, i.e., data-driven. The six-phase guide to conducting TA, as outlined by Braun et al. [42], was employed:

- Familiarisation with the data
- Generating initial codes
- Organisation of the initial codes into patterns to generate themes
- Reviewing themes (checking themes against raw data to ensure a good fit and reclassification of themes into levels)
- Defining and naming themes
- Interpretation

Trustworthiness was enhanced using three methods. Firstly, all transcripts were independently coded by all three members of the research team, and the codes were compared. This supported validation of codes, supporting valid theme development. Theme development was a collaborative process involving all three researchers meeting together to discuss themes. Secondly, attention was given to negative cases, which allowed for discussion of different perspectives and contradictions in the data. Analysis of negative cases helped to refine the interpretation of the data. Finally, reflexive strategies were used to reduce the risk of researcher bias. This was particularly important as the research team had prior knowledge and expertise in leadership and management, and workforce development. Two reflexive strategies were used: (i) “oppositional arrangement of perspectives” supports researchers to become aware of the range of perspectives at work within established frames of social norms [43] and (ii) “backgrounding” whereby “background” data (data that on first analysis may seem less significant) are foregrounded. The transformation from background to foreground prompts researchers to investigate whether any topics of potential significance that had not been expected were encompassed within the text [43]. Member checking by sending draft codes and themes to participants was attempted. However, none of the participants were able to respond, as they generally felt that time constraints due to COVID-19 made responding difficult.

## 4. Results

Findings from the thematic analysis revealed three key themes representing the staff's experiences of leadership: (i) defining leadership, (ii) opportunities for leadership, and (iii) promoting leadership development. Each of these and interview evidence supporting them will be presented in turn.

### 4.1. Theme 1: Defining Leadership

Participants struggled to agree on a clear definition of leadership in the context of nursing; in particular, there was some tension about whether nature/innate ability or nurture was most critical to leadership development. This respondent felt that leadership was contingent on experience; i.e., it was nurtured over time:

*P1: …if there's no manager or sister then the most experienced nurse on the ward [takes charge]*.

One participant felt leadership was contingent on the combination of nurture/nature:

*P4: I think it's a product of your experience, but unless you've got the inherent qualities you can't just create a leader*.

However, the general consensus was that leadership relied on nature/innate qualities, rather than a set of qualities that could be nurtured:

*P6: …it's about having a natural trait*.

*P9: …unless you've got the inherent qualities you can't just create a leader*.

These comments are interesting for their emphasis on leadership as primarily innate and an inherited trait, rather than universal, and how this appears to run counter to recent directives calling for all nurses to fulfil the requirement to develop leadership skills which assume it is a universal quality.

Participants also associated leadership with a higher level of seniority, beyond their own band 5 status and entrenched within the organisational, hierarchical structure:

*P2: [Leadership is] The ward manager, the sisters, the band 6's*.

*P12: It's your senior staff-your Band 6s and 7s, Matrons and definitely the nurse-in-charge on that shift*.

Despite this, some participants recognised that although (as band 5 nurses) they wielded less authority compared with higher bands, they nevertheless carried out a leadership role:

*P3: My experiences are…right from a band 5 you're leading because you're delegating tasks [to health care assistants]…finding out who is competent to do those skills*.

Participants, therefore, associated leadership with seniority/delegating responsibility to others, requiring a sound knowledge of other staff's competencies.

Despite the apparent majority view that leadership relies on inherent qualities, other evidence appeared to contradict this; i.e., a number of participants felt in nursing it relied on nurture/experience as opposed to natural talent:

*P13: I don't think people take you seriously to do leadership when you're a little bit younger…*

Moreover, this view seemed to present a bias as well as a barrier for younger staff who might aspire to lead:

*P6: I find it hard to lead someone who is older or been on the job longer*.

Despite these tensions/contradictions, there was consensus among participants that as band 5 nurses they may not specialise in leading staff, but they nevertheless specialised in leading care and participants acknowledged this important distinction:

*P11: I am a leader of my own care for my own patients*.

*P1: Leading patient care, being in control of your own pod…*

Taking leadership/responsibility for patient care was generally viewed as separate and discrete from leading staff but certainly not inferior to it:

*P9: I think leading patient care, to me, is the most important part of leadership.*

*P3: [As leaders] we all have responsibility to strive and deliver best outcomes for patients*.

While promotion beyond band 5 was perceived to increase opportunities to lead/manage others, it was also viewed by several participants as shifting away from leading patient care; something, they were opposed to:

*P5: I don't want to lose patient contact and still be a nurse. I wouldn't want to be a band 6 because of the loss of patient contact*.

Despite some reservations, the majority of respondents felt band 6 offered the best compromise between allowing nurses to continue to lead care while also leading staff. However, promotion to band 7 was perceived by many to denote the demarcation point at which directly leading patient care abruptly ended:

*P2: The idea of being a sister is great, but I don't want my day monopolised by doing rotas/staffing. I want patient contact*.

Furthermore, participants perceived band 7 as a point of departure from close, collegiate working towards a more distal, managerial role:

*P11: I think we've got one Band 7 in the building. We rarely see her.*

*P11: I've never met a Matron*.

While a range of perceptions, some contradictory, were uncovered regarding how band 5 nurses viewed leadership, greater consensus was found concerning leadership style. In general, participants' nurses preferred more democratic leadership styles, especially their perceived capacity to offer motivation/inspiration/mutual respect:

*P5: Leadership is a person who you can always turn to who knows the answers but who will talk to you in a way that is encouraging… [not] in a derogatory fashion*.

*P10: I know my band 6, I can ask her anything…She just rings me all the time and tries to motivate me.*

By contrast, autocratic leadership styles were generally viewed negatively:

*P5: Very, very dominant. “I am the boss! This is how it's going to be!” I feel like she's very strict*.

This participant highlighted how the autocratic leadership style discouraged close, collegial working, even where nurses demonstrated high levels of competency, inducing feelings of negativity:

*P1: So, even if you might be a good nurse, I think, “well they're not going to like me as a person.” Even if I do the job well…and that's disheartening*.

By contrast, more democratic leadership styles were perceived in a positive light and as providing good guidance, especially where there was more an exchange of knowledge (transactional leadership) as opposed to a more didactic approach/style:

*P13: [Leaders]…give clinical guidelines, policies and procedures for effective practice based on the knowledge and NICE guidelines as well, so they become our standard operating procedures to guide us*.

A notable finding also was that transactional style was more likely to be evidenced in very acute care settings such as emergency departments, theatres, and critical care wards. This suggests that leadership style is not only determined by individual choice but also by context/setting which may be instrumental in facilitating (or impeding) certain styles. One explanation for the salience of transactional style found here in emergency departments/theatres/critical care wards may be that these settings require rapid, expert decision-making, and timely adherence to protocols that make good guidance/effective knowledge exchange a priority.

Moreover, leadership style may not only be influenced by context/setting but more specifically still the unique circumstances currently unfolding within that context/setting. This means leadership style may be more fluid than fixed:

*P7: [He is] a democratic leader, but he can be a transactional leader and a rational leader*.

In summary, band 5 nurses' perceptions of leadership and how it is best defined revealed a range of interpretations that were sometimes contradictory. Leadership in the context of nursing appears to be nuanced, complex, and variable and influenced by multiple factors, rather than representing a fixed/immutable concept that is easily pinned down.

### 4.2. Theme 2: Opportunities to Lead

Discussing band 5 leadership, many participants indicated their experience of leading people as a hallmark of leadership (as distinct from leading patient care) was determined by *opportunities* to lead. This appeared very much contingent on work setting/working arrangements/practical necessity, rather than strategic or planned professional development. For example, participants who worked in care homes highlighted that they were often the only registrant nurse on their unit and therefore automatically expected to lead in terms of staffing/resources/resident care. This expectation fed into nurses' perception leadership was integral to this particular role:

*P1: I think for each care home the nurse is like a leader. You need to take responsibility of your floor and your staff…*

Similarly, participants working on wards in secondary care revealed opportunities to take charge of their ward that entailed leading both staff and patient care:

*P12: There's always a nurse in charge for the shift…you're still in charge of the full 27 patients as well as the staff*.

Opportunities for band 5 nurses to lead were especially prevalent where senior staff were unavailable and, of practical necessity, responsibility for leadership was delegated:

*P3: You're leading the whole ward when your manager is not there*.

Respondents working on wards in secondary care welcomed opportunities to take on a stronger leadership role, but some would like more frequent opportunities:

*P1: I would like to get some more experience of being the nurse in charge of the ward when the sisters aren't there*.

*P13: I would really like to be the nurse in charge and get my skills…*

Participants from the emergency department also reported opportunities to lead, though this tended to be limited to a lower level of leadership:

*P13: I would normally lead on the floor…I will look after…health carers or a junior member of staff…the majority of the time…*

By contrast, participants working in ITU settings indicated there were generally few opportunities for leadership. One respondent revealed that in their area of practice, band 5 staff is actively discouraged from leading:

*P6: I am…more encouraged to stay at the bedside and try to limit my job to…doing observations-not expressing leadership skills*.

Care setting also limited opportunities to lead where participants worked in the community. Although band 5 nurses were assigned case-loads conferring much responsibility, they primarily operated as lone workers that restricted them to leading patient care:

*P14: I am a leader of my own care for my own patients*.

A key finding in general was that although band 5 nurses had ample opportunity to lead patient care, opportunity to lead people was highly variable and contingent on context/circumstances. Some participants were seldom offered the opportunity to lead people, while others were comparatively overwhelmed with opportunities. Often, this meant an imbalance regarding opportunities to lead people, as highlighted by these participants working in theatres and EAU who often faced emergency situations:

P7: . . .a band 6 as team leader. . .rang in sick so I was moved up as the team leader. . .It was quite a struggle, obviously. I said. . .“it's not my choice to be the team leader, I was put here and I'll play that role, but if something happens, it's my name they're going to. . .chase up.”

P14: We went on shift one night‐it was me and another junior member of staff. . .we were left in charge of the patient services as well because they'd gone off sick and no one could cover. So, we were in charge of bed management in the hospital, as well as running the suite. . .I was nervous. . .

While more experienced band 5 nurses may be very capable of taking on intensive leadership roles, there needs to be a choice, rather than an expectation of compliance. Such decisions should always place patient safety first. Band 5 nurses made a clear distinction between leading care and leading people with opportunities to develop the latter being highly variable and contingent on individual context/circumstances.

### 4.3. Theme 3: Promoting Leadership Development

Responses indicated a paucity of formal development via bespoke training/development programmes. In lieu, all participants reported heavy reliance on *informal* development that could be limited to being self-directed/opportunistic, i.e., observing colleagues, rather than planned or more deliberately co-ordinated:

*P3: I think from being a Band 5 I might not necessarily have done a course, but I've reflected…It's about observing leaders. Picking bits of how you would want to be and how you don't want to be develops me*.

*P12: I think shadowing and mirroring are how I've learned. I wouldn't necessarily say that anyone has taken me aside and taught me anything…when I've led, I've picked up on a lot of things that I've seen other people do*.

However, that informal training can be effective is illustrated here:

*P14: I get the most out of the time I've shadowed “X” who is the band 6 because she is making all of these decisions…she's explaining how processes work, she'll go through anything with me and explain why she's made the decision. She's got all the qualities of a leader…*

Where informal training seemed particularly effective was when senior staff demonstrated a transformational leadership style that inspired trust, motivation, and empowerment in band 5 nurses:

*P13: …if I work alongside the Band 7's they are good at developing us or empowering us to do some leadership assignment. Usually, this goes with the patient care itself, like managing the zone and…I can perceive a sense of empowerment. That they're trying to input encouragement for me*.

While the opportunity to shadow/mirror good leadership and receive encouragement/opportunities to develop it may be vital, arguably band 5 leadership should also be encouraged through formal leadership training as an adjunct to this:

*P3: Watching and learning-that helps develop me as a leader. That's a challenge I would give myself. I would read some reading material…like if there's a new [leadership] practice of interest to me…*

Participants also perceived how the provision of formal leadership training could benefit not only themselves but also the team and the wider organisation:

*P3: [Formal leadership]…to actually understand with theory how you put that into practice. How the theory can make you a better leader. How this will make you more insightful…to develop myself, to develop the service, to develop others*.

Despite respondents' enthusiasm for formal leadership training, several band 5 nurses reported difficulties accessing it and even open discouragement:

*P6: They don't like that when I'm doing that [requesting leadership development]. They always try to keep me in the corner. So, it's very difficult to express or maybe to learn how to be a leader*.

*P8: …they don't really emphasise leadership when you're a Band 5*

*P11: If I ask for leadership development I will get laughed at*.

These examples illustrate how band 5 nurses may often be motivated/dedicated to the formal development of leadership skills, in accordance with guidelines recommending this (e.g., [1]), but can face systemic bias that prevents them. Moreover, this systemic bias appears to persist even among band 5's employed in settings where good leadership is essential to their role:

*P7: …we're team leaders for night shifts, but we didn't get the formal training of what being a team leader is, what qualities you need to lead a team*.

Even where an exception was found and one participant initially granted support for formal leadership training, the promised support was unforthcoming:

*P8: I'm doing the Nightingale course so I'm meant to have a 1: 1 mentor and to actually do some shadowing and things with them, but that hasn't happened…*

Moreover, the Florence Nightingale Leadership Development Course represents one of only a handful of formal leadership courses currently available to band 5 nurses in England.

In summary, band 5 nurses valued good quality informal leadership training that included shadowing/mirroring. This could be facilitated by the senior staff's adoption of a transformational style [44]. However, informal training could be limited to being self-directed/opportunistic, rather than planned/more deliberately co-ordinated. Opportunities for formal training were limited/not fully supported.

## 5. Discussion

Thematic analysis of the data elicited several key findings, and a robust attempt was made to ensure proportionality between the data and the analytical claims and conclusions presented in this discussion.

Consistent with previous findings (e.g., [28, 45]), this study found a lack of consensus regarding how band 5 nurses perceived “leadership” in the context of nursing. While one participant held the view that leadership is created by an amalgam of nature/nurture, others felt that strong leadership required a particular set of innate qualities closely intertwined with personality traits. This latter view runs counter to recent evidence that traditional trait or personality approaches are of little value when it comes to delivering leadership training (e.g., [16]). Contradictions were also found in this study; e.g., while many respondents perceived leadership to be an innate quality, they also felt entitlement to lead was based on nurture/experience, rather than innate abilities/talents. This also appeared to present a bias against younger staff leading or aspiring to lead. These comments are interesting for the way they appear to run counter to recent directives calling for *all* nurses to fulfil the requirement to develop leadership skills (e.g., [1]) that carry the assumption all nurses are capable of leading both care and people.

Leadership was most frequently associated with hierarchical staffing structures and “top down,” from the management level to the staff nurse on the ward and particularly the managers and those who held band 7 and band 6 positions. This required the ability to delegate responsibility to others and sound knowledge of other staff's competencies. Notably, however, the majority of participants felt leadership was associated with higher seniority, *beyond* band 5, and therefore largely outside their domain. There was consensus that band 5 nurses had a leadership role in terms of leading *patient care*. Notably, though, leading care was perceived to be quite different and discrete from “leadership of other people.” Participants perceived “leading care” to be as vital as “leading people,” if not more so, but viewed them as mutually exclusive. A few band 5 nurses recognised that although they wielded less authority, they carried out a leadership role-albeit at a different, often lower hierarchical level. Taken together though, the findings support Stanley's [22] contention that more work is needed to outline what clinical nurse leaders are, and how they can recognise themselves as leaders.

The findings further reveal that band 5 nurses strongly perceive they work within a fairly rigid hierarchy in which clear divisions exist between themselves as subordinates on one side and leaders/management on the other in which “leadership” was frequently perceived to be synonymous with “management,” especially beyond band 6. As Stanley [46] emphasises, “leadership” and “management” are not interchangeable concepts and there is danger in using them indiscriminately to delineate roles within healthcare systems. Management is primarily concerned with assuming control and maintaining a clear division between workers/patients [47], and this should not be conflated with leadership in healthcare which should ideally involve a more closely bound relationship between staff [48] and also patients [24]. Adherence to older 20th-century management models that promote hierarchical ordering of staff in which leaders and “subordinates” work in separate spheres have come under criticism for being outmoded/ineffective [49], particularly where they continue to be applied within the NHS [9].

A related finding was band 5 nurses' perceptions that promotion automatically signals a shift towards management and a concomitant move away from nurses' principal vocation to provide the patient care in close collaboration with colleagues. Such perceptions potentially presented serious barriers to seeking promotion or fulfilling aspirations towards leadership. These perceptions appeared to be reinforced when senior staff adopted more autocratic leadership styles consistent with more rigid/hierarchical ways of working, and the more arcane management models discussed earlier.

Greater consensus was found regarding band 5 nurses' perceptions of what constituted optimal leadership style where high value was placed on senior staff's adoption of more democratic styles. Regarding this, the transformational style of leadership was valued highly, especially where it was manifest in senior staff's adoption of an approachable, motivating and reassuring stance that presented a good role model to follow and inspired trust [44]. As Gopee and Galloway [50] note, effective leaders tend to focus on influencing staff's behaviours and especially motivating, inspiring and energising individuals. It is not only how band 5 nurses perceive/define leadership that may be important but also the example/influence set, reflected in senior staff's leadership style. By contrast, autocratic leadership styles were perceived negatively.

Of note, this study found evidence that senior staff who promoted knowledge exchange in an approachable and collaborative way were more likely to be found in very acute care settings such as emergency departments, theatres, and critical care wards. This perhaps reflects the need in such settings for rapid, expert decision-making, ensuring the team closely adheres to set protocols in a timely manner. This finding underscores the importance not only of leadership style but also context/setting in providing ideal environments for certain styles to flourish. Participants working on wards in secondary care similarly reported opportunities to take charge of their ward and lead staff and patient care. Nevertheless, there needs to be a balance. Some band 5 nurses expressed concerns they sometimes felt overwhelmed with opportunities to lead and out of their depth. Arguably, there is a requirement for training in leadership in advance that should accompany opportunities to lead.

Meanwhile, band 5 nurses who worked in care homes were often the only registrant nurse on their unit and automatically expected to lead in terms of staffing, resources, and resident care. This expectation-reinforced nurses' perception leadership was integral to their role but not necessarily supported by specific leadership training.

By contrast, participants working in ITU settings indicated that, in general, there were limited opportunities for leadership with one participant reporting active resistance to this by senior staff. Similarly, band 5 nurses working in community settings rarely encountered opportunities to lead due to their role as lone workers.

Evidence was found for participants' motivation to acquire leadership skills. However, while this could be encouraged where senior staff adopted more democratic leadership styles (e.g., transactional or transformational styles), opportunities for leadership development were frequently found to be contingent on conducive care contexts/settings which were only available to some band 5 nurses. Moreover, leadership development trended to be limited to being self-directed and opportunistic, rather than planned and deliberately co-ordinated.

This study highlights how both these factors make access to informal leadership development by band 5 nurses highly variable and not always equitable. This inequity was likely to be exacerbated where there was a lack of support for band 5 nurses' *formal* leadership training, which this study also uncovered. This is a situation also made problematic by the paucity of training programmes currently available in England.

One way to restore equity/increase opportunities for leadership for *all* nurses would be the creation of nationally available, formally validated leadership development programmes dedicated to promoting *leadership of people* as distinct from leading care and bespoke/available to all band 5 nurses who wanted it, irrespective of age. Programmes should ideally focus on developing clinical leadership skills and potential, as distinct from managerial skills training and complement rather than detract from leading good patient care. Ideally also, training should be resourced and overseen by an external governing body to ensure that it combines formal training supported by mentors with ample opportunities for practice-based training alongside informal shadowing/mirroring of good practice as an important adjunct rather than a replacement to this.

### 5.1. Limitations

This was a relatively small-scale cross-sectional study involving 18 participants from North East England. The response rate was lower than planned due to the outbreak of COVID-19 which impacted on the availability of primary care and voluntary sector staff. Further research is needed in this area, including consultation with larger samples from other regions and a wider range of clinical settings to establish how band 5 leadership training could be better designed, resourced and implemented, and externally governed and validated.

## 6. Conclusions

This study explored band 5 nurses' perceptions of leadership across a diverse range of different healthcare contexts/settings in England. This revealed a number of deficiencies and inconsistencies regarding how leadership is defined, characterised, and promoted. Ways of improving quality and provision are discussed. Given high rates of senior staff attrition in England and recent guidelines promoting nurse leadership development at *all* levels as a prerequisite to high quality, safe, and compassionate health care, there is a burgeoning need for renewed discussion of band 5 nurses' leadership development.

## Figures and Tables

**Table 1:** Participants' characteristics.

Care provider organisation	Care service location	Care service	Participant	Gender	Age	Years qualified	Country of birth
NHS trust 1	Hospital 1	Surgical ward	P1	Female	20–29	<1 year	UK
NHS trust 1	Hospital 1	Medical ward	P2	Female	20–29	<1 year	UK
NHS trust 1	Hospital 1	Emergency department	P3	Male	20–29	6–10 years	UK
NHS trust 1	Hospital 1	Critical care	P4	Female	30–39	3–5 years	UK
NHS trust 1	Hospital 1	Emergency department	P5	Male	40–49	10–15 years	Philippines
NHS trust 1	Hospital 2	Medical ward	P6	Female	20–29	1-2 years	UK
NHS trust 1	Hospital 2	Theatres	P7	Male	30–39	3–5 years	UK
NHS trust 1	Hospital 2	Theatres	P8	Female	30–39	3–5 years	UK
NHS trust 2	Hospital 1	Medical ward	P9	Female	40–49	1-2 years	UK
NHS trust 2	Hospital 1	Medical ward	P10	Female	40–49	6–10 years	UK
NHS trust 2	Hospital 2	ITU	P11	Female	40–49	21–25 years	Czech Republic
NHS trust 2	Hospital 2	Theatres	P12	Male	50–59	2–5 years	Philippines
NHS trust 2	Hospital 2	ITU	P13	Female	20–29	<1 year	UK
NHS trust 2	Hospital 2	Admissions unit	P14	Female	30–39	<1 year	UK
NHS trust 3	Community 1	Community	P15	Female	20–29	<1 year	UK
Care home company 1	Care home 1	Care home for older people	P16	Female	30–39	1-2 years	UK
Care home company 2	Care home 1	Care home for older people	P17	Female	30–39	3–5 years	Romania
Care home company 3	Care home 1	Care home for older people	P18	Female	60–69	>30 years	UK

## Data Availability

The data supporting the current study are available from the corresponding author upon request.
